# Locally Advanced Breast Implant-Associated Anaplastic Large-Cell Lymphoma: A Case Report of Successful Treatment with Radiation and Chemotherapy

**DOI:** 10.3389/fonc.2015.00026

**Published:** 2015-02-18

**Authors:** Christopher F. Estes, Da Zhang, Ruben Reyes, Richard Korentager, Marilee McGinness, Christopher Lominska

**Affiliations:** ^1^Department of Radiation Oncology, University of Kansas Medical Center, Kansas City, KS, USA; ^2^Department of Pathology, University of Kansas Medical Center, Kansas City, KS, USA; ^3^Department of Hematology, University of Kansas Medical Center, Kansas City, KS, USA; ^4^Department of Plastic Surgery, University of Kansas Medical Center, Kansas City, KS, USA; ^5^Department of Breast Surgery, University of Kansas Medical Center, Kansas City, KS, USA

**Keywords:** breast implant-associated anaplastic large-cell lymphoma, breast, breast implant, non-Hodgkin lymphoma, radiation therapy, chemotherapy

## Abstract

The development of breast implant-associated anaplastic large-cell lymphoma (ALCL) is a rare phenomenon. A typical presentation is an effusion associated with a breast implant. Less commonly, disease can be more advanced locoregionally or distantly. The optimal treatment schema is a topic of debate: localized ALCL can potentially be cured with implant removal alone, while other cases in the literature, including those that are more advanced, have been treated with varying combinations of surgery, chemotherapy, and external beam radiotherapy. This is a case report of breast implant ALCL with pathologically proven lymph node involvement, the fifth such patient reported. Our patient experienced a favorable outcome with radiation therapy and chemotherapy.

## Introduction

**Figure 1:**
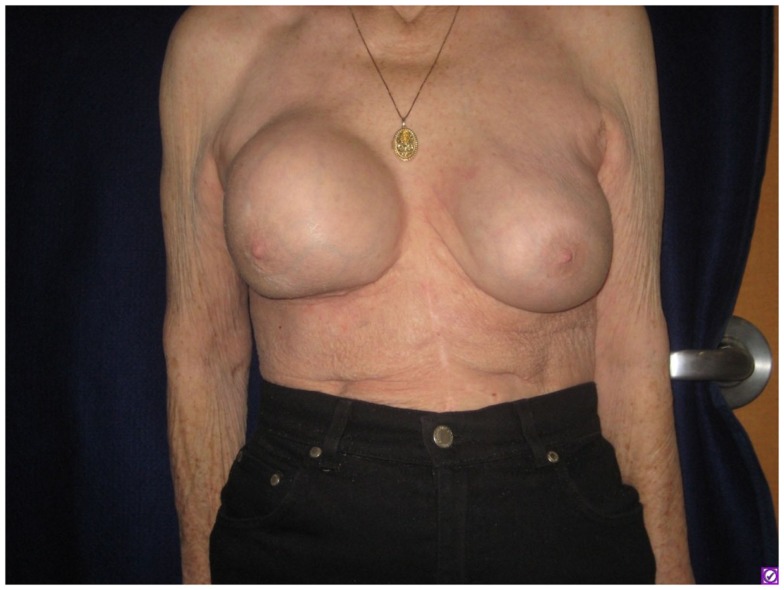
**Firmness and marked increase in size of the right breast as seen on presenting physical examination**.

A 76-year-old Caucasian woman was found to have implant-associated anaplastic lymphoma with pathologically proven nodal involvement and was successfully treated by implant removal, chemotherapy, and radiotherapy. She had gel breast implantation performed 20 years prior to presentation. These were later replaced by saline implants. One year before presentation the saline implants were leaked and replaced with gel implants. She developed a recurrent fluid collection involving her right breast (Figure 1). A drain was placed and yielded minimal output before being removed 1 week later. Cytology of the fluid demonstrated atypical appearing lymphocytes. The fluid later reaccumulated, and right axillary lymphadenopathy was noted on physical exam (largest node 5.1 cm on ultrasound). Core needle biopsy of the node revealed rare atypical cells.

**Figure 2:**
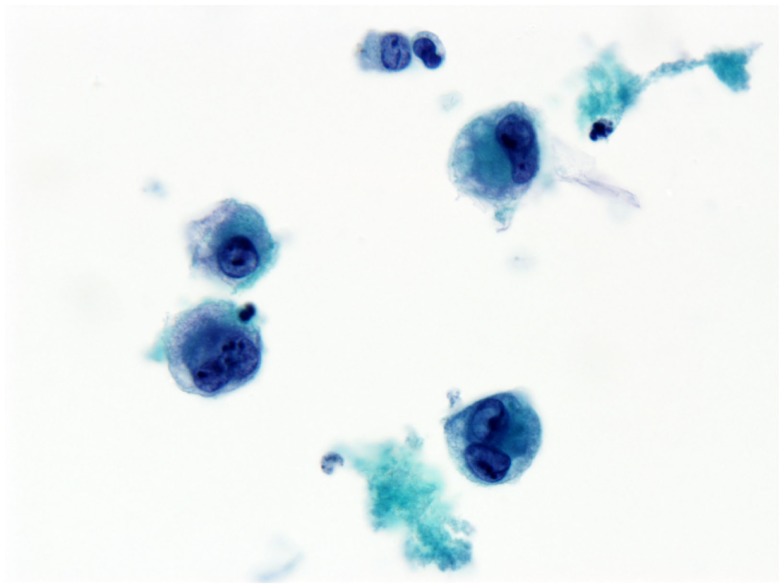
**Effusion fluid (seroma)**. Anaplastic large cells present in the effusion surrounding the breast implant. Anaplastic large cells also involve the breast implant capsule (not shown). Immunohistochemical staining shows anaplastic large-cell lymphoma T-cell type.

**Figure 3:**
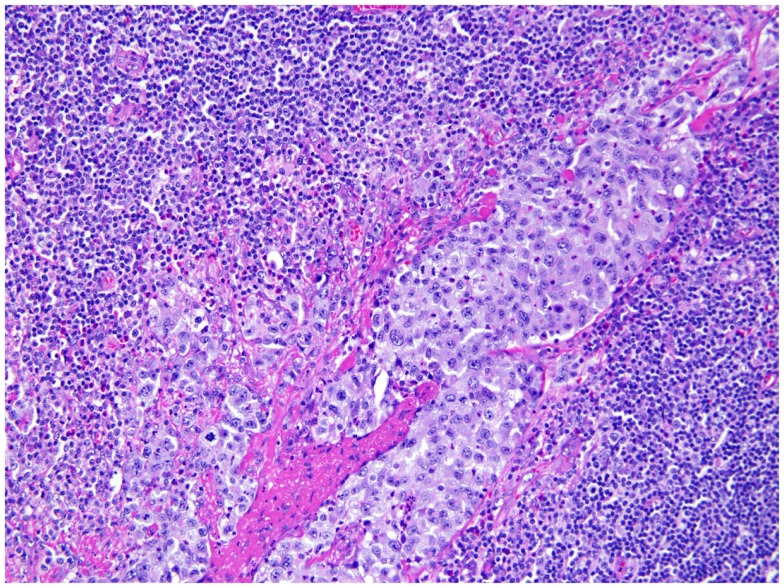
**Axillary lymph node**. Axillary lymph node of the same patient shows large atypical cells involving the sinusoidal and lymphatic space, consistent with anaplastic large cells involving the lymph node.

**Figure 4:**
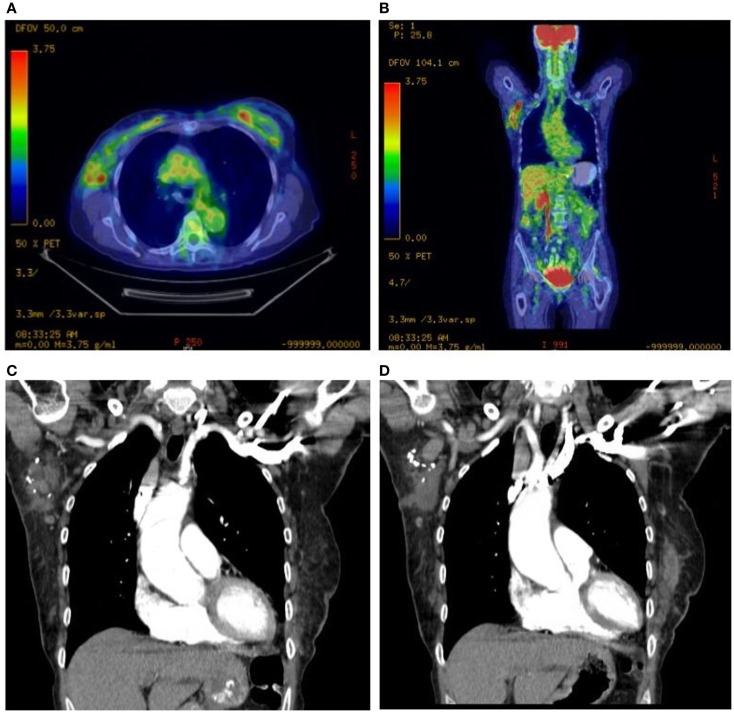
**Right axillary lymphadenopathy demonstrated on axial and coronal PET scan images with abnormal FDG avidity (A,B) and coronal CT images with contrast (C,D)**.

She underwent capsulectomy and right axillary nodal excisional biopsy with bilateral implant removal in 2011. On histopathologic analysis, anaplastic large-cell lymphoma (ALCL), anaplastic lymphoma kinase (ALK)-negative was demonstrated in the fibrous capsule, cystic fluid, and axillary lymph nodes (Figures 2 and 3). Imaging with CT and PET scans demonstrated residual right axillary lymphadenopathy with FDG avidity, as illustrated in Figure 4. Bone marrow analysis was performed and showed no evidence of lymphoma involvement. The patient was staged as Ann Arbor stage IIE. She was treated with six cycles of cyclophosphamide 750 mg/m^2^, doxorubicin 50 mg/m^2^, vincristine 2 mg, and prednisone. Prednisone required a dose reduction from 100 to 75 mg after the first cycle to minimize hyperglycemia secondary to diabetes mellitus type II. Pegfilgrastim 6 mg was injected each cycle for hematopoietic support. Ciprofloxacin 500 mg BID was used daily for bacterial infection prophylaxis. After cycle 2, she began to display adverse effects of nausea grade 2, oral mucositis grade 1, and dysgeusia grade 2 per National Cancer Institute Common Terminology Criteria for Adverse Events grading scale version 4.03 (1). Tetrahydrocannabinol was administered for treatment of nausea and anorexia. Weight loss of grade 1 occurred by the end of therapy. Interval PET and CT imaging were performed after cycle 2, demonstrating metabolic activity of SUV 0.88 in the right axillary lymph nodes, a partial response. Repeat PET imaging was performed after cycle 4 of chemotherapy, confirming complete response to treatment.

**Figure 5:**
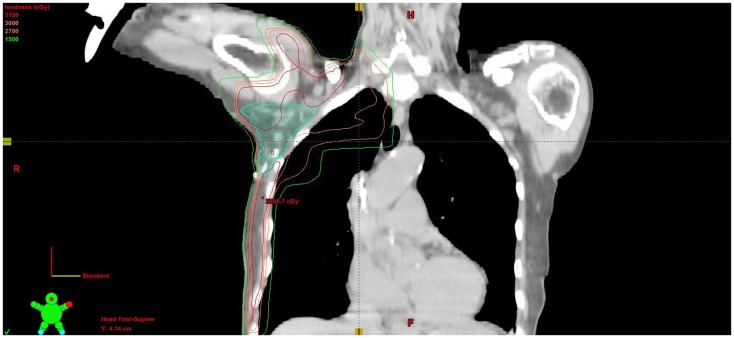
**Coronal CT slice of treatment plan showing isodose lines and contoured axillary nodal volume**.

Adjuvant radiation therapy was then delivered to the right breast, axilla, and right supraclavicular nodes to 30.6 Gy in 1.80 Gy fractions, finishing in January of 2012. A monoisocentric technique was used with breast tangents matched to a field covering the supraclavicular nodes and high axilla. The axilla levels 1–3 were contoured and a lightly weighted posterior axillary field was used to bring the volume up to the prescription dose (Figure 5). With therapy, the patient experienced grade 1 dermatitis manifested as faint erythema within the treatment field. Two-year follow-up with PET imaging and clinical evaluation showed no evidence of disease. The patient wished to have breast augmentation again. She underwent silicone gel implant placement bilaterally and has been followed for an additional year without evidence of disease.

## Background

Primary breast lymphomas represent about 0.1–0.5% of total breast tumors, and approximately 3% of non-Hodgkin lymphomas of extranodal sites (2). Tumors of ALCL histology represent 2% of adult non-Hodgkin lymphomas and are the second most common peripheral T cell lymphoma (3, 4). The entity of breast implant-associated ALCL was first described by Keech and Creech (5) in 1997, and currently at least 60 cases have been described in the literature (6). A study in the Netherlands estimated that the incidence of breast implant-associated ALCL is 1 out of 500,000 per woman with prostheses per year, using a case–control model with breast lymphomas other than ALCL as the control (7). However, there has been controversy regarding the level of risk associated with breast prosthesis implantation (8). The FDA reports that 5–10 million women have undergone breast implantation, leading to a possible estimation of breast-associated ALCL incidence of 1:80,000 to 1:170,000 given the 60 reported cases thus far (9).

Morphologically, ALCL is composed of large blastic cells with pleomorphic, often horseshoe-shaped or multiple nuclei. Growth is in a cohesive pattern often involving lymph node sinuses with a variable composition of granulocytes and macrophages. In breast-associated and cutaneous ALCL, the cells are CD30 positive on cytogenetic studies and negative for ALK (10). In contrast, systemic ALCL is characterized by positive ALK (11). Those who present with a mass rather than an effusion have a higher risk of failure or relapse (12). Various treatment regimens have been employed including surgery (implant removal) followed by observation, chemotherapy alone, chemotherapy and radiation, and radiation alone. Patients who present with disease limited to an effusion surrounding the fibrous capsule surrounding the implant have been shown to have a favorable outcome with observation; however, there are rare cases of advanced and treatment-resistant disease leading to fatality (13). The ideal treatment schema is yet to be elucidated.

We report a locally advanced case of breast implant-associated ALCL at our institution. Pathologic axillary nodal involvement by breast implant-associated ALCL is rare: only four cases of pathologically confirmed disease of the axillary nodes have been described. Of the 60 cases reported, workup for axillary nodal involvement was carried out in 29, 10 of which were found to have clinical evidence of axillary lymphadenopathy. Four of these were confirmed pathologically (6); our patient represents the fifth.

## Discussion

Treatment methods in breast implant-associated ALCL have included surgery, radiation therapy, chemotherapy, their various combinations, and observation. Overall survival is typically favorable, and only three patients have been reported to die of disease. ALCL can also present in a systemic or cutaneous manner. Systemic disease is typically more aggressive; those with cutaneous lymphomas carry a more favorable prognosis and negative ALK, similar to breast ALCL (14). On long-term follow-up of the 60 patients reported by Miranda et al., patients who were found to have disease confined to the fibrous capsule experienced improved complete response, overall survival, and progression-free survival. Those treated with chemotherapy alone did not experience improved outcome compared to those treated with either watchful waiting or radiotherapy alone. The four patients who received radiotherapy alone are free from recurrence (6).

Patients who present with effusion confined disease have had long-term relapse free intervals with observation alone. Those with a mass show a more aggressive prognosis, and may achieve a greater benefit with combined modality therapy with chemotherapy and radiation. Implant removal in ALCL is recommended: in one review, three of four patients without implant removal relapsed, all of whom responded to salvage therapy (12). More aggressive tumors may arise in those patients with prior breast cancer (15).

## Concluding Remarks

For the advanced presentation of axillary nodal involvement in our patient, we obtained a favorable outcome with combined modality treatment with radiation therapy and chemotherapy. Our patient chose to undergo placement of new implants after her treatment. It was discussed with the patient that the risks of implant replacement after treatment are unknown in this setting. She chose to proceed and has had no adverse consequence to date. Finally, this clinical scenario highlights the importance of regional nodal evaluation in patients with breast implant ALCL.

## Conflict of Interest Statement

The authors declare that the research was conducted in the absence of any commercial or financial relationships that could be construed as a potential conflict of interest.
